# Educational step-by-step surgical video about operative technique in robotic pancreaticoduodenectomy (RPD) at University of Illinois at Chicago (UIC): 17 steps standardized technique—Lessons learned since the first worldwide RPD performed in the year 2001

**DOI:** 10.1007/s00464-020-07383-0

**Published:** 2020-01-17

**Authors:** Pier Cristoforo Giulianotti, Alberto Mangano, Roberto E. Bustos, Eduardo Fernandes, Mario A. Masrur, Valentina Valle, Antonio Gangemi, Francesco M. Bianco

**Affiliations:** Division of General, Minimally Invasive and Robotic Surgery, University of Illinois at Chicago, 840 S Wood Street, Suite 435 E, Clinical Sciences Building, Chicago, IL 60612 USA

**Keywords:** Robotic pancreatoduodenectomy, Whipple procedure, Evidence based surgery, Pancreatic surgery, Pancreatic cancer, Minimally invasive surgery

## Abstract

**Background:**

RPD (Robotic pancreatoduodenectomy) was first performed by P. C. Giulianotti in 2001 (Arch Surg 138(7):777–784, 2003). Since then, the complexity and lack of technique standardization has slowed down its widespread utilization. RPD has been increasingly adopted worldwide and in few centres is the preferred apporached approach by certain surgeons. Some large retrospective series are available and data seem to indicate that RPD is safe/feasible, and a valid alternative to the classic open Whipple. Our group has recently described a standardized 17 steps approach to RPD (Giulianotti et al. Surg Endosc 32(10): 4329–4336, 2018). Herin, we present an educational step-by-step surgical video with short technical/operative description to visually exemplify the RPD 17 steps technique.

**Methods:**

The current project has been approved by our local Institutional Review Board (IRB). We edited a step-by-step video guidance of our RPD standardized technique. The data/video images were collected from a retrospective analysis of a prospectively collected database (IRB approved). The narration and the images describe hands-on operative “tips and tricks” to facilitate the learning/teaching/evaluation process.

**Results:**

Each of the 17 surgical steps is visually represented and explained to help the in-depth understanding of the relevant surgical anatomy and the specific operative technique.

**Conclusions:**

Educational videos descriptions like the one herein presented are a valid learning/teaching tool to implement standardized surgical approaches. Standardization is a crucial component of the learning curve. This approach can create more objective and reproducible data which might be more reliably assessed/compared across institutions and by different surgeons. Promising results are arising from several centers about RPD. However, RPD as gold standard-approach is still a matter of debate. Randomized-controlled studies (RCT) are required to better validate the precise role of RPD.

**Electronic supplementary material:**

The online version of this article (10.1007/s00464-020-07383-0) contains supplementary material, which is available to authorized users.

The current short description with video visually illustrates and it is complementary to the technique previously described in the paper published by our group in 2018 [1]. Herein, we epitomize the 17 surgical steps as a complementary adjunct to the associated surgical educational video.

## Methods

The current project has been approved by our local Institutional Review Board (IRB). We edited a step-by-step video-guidance of our RPD standardized technique. The data/video images (which are exclusively intraoperative/intrabdominal with the patient information not identifiable) were collected from a retrospective analysis of a prospectively collected database (IRB approved). The narration and the images describe hands-on operative “tips and tricks” to facilitate the learning/teaching/evaluation process. Written consent has been obtained prior to the operation.

### RPD surgical technique

The trocars placement, the robotic docking details and additional complementary details of all the surgical steps are in-depth described in our previous article [1].

**Fig. 1:**
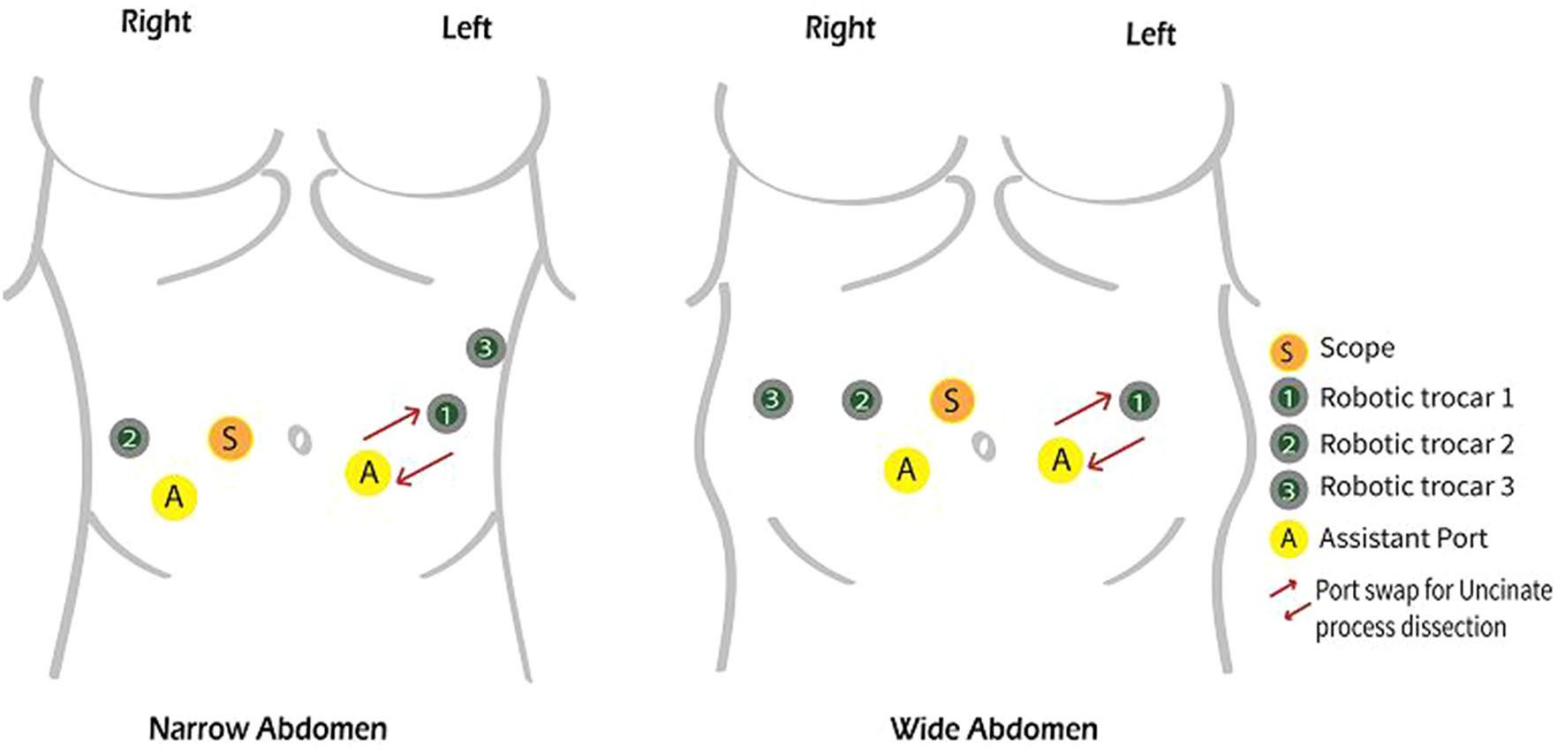
Port placement

However, trocar placement is paramount for setting the operation up for sucess and a properly standardized port setting will ease the operative surgical steps. Herein, we provide an epitomized guide to trocars placement with a visual represention shown in Fig. 1. Additionnally, our port setting variations in accordance to the patient’s *habitus* are also shown in Fig. 1. The patient is positioned on a bean-bag in supine positon with upper limbs tucked and split legs in French position [1]. The table is slightly tilted towards the left and in a 20° reverse Trendelenburg. The assistant surgeon stands between the patient’s legs. The pneumoperitoneum is achieved with Veress technique [1]. A 5 mm port is placed in the left-upper-quadrant (LUQ) and used for a preliminary diagnostic laparoscopy to carry out detailed staging of the disease. Trocar placement is the essential preliminary operative step to set the operation up for success [1]. We have shown our port setting approach in Fig. 1, indicating variations in accordance to the patient’s *habitus* [1]*.* Should the Si-HD system be used, the robotic cart has to be docked head-on. A 12 mm scope is positioned along the right para-rectal line at its crossing with the transverse-umbilical-line [1]. In such a location, the camera allows improved vision of the pancreatic uncinate process, portal vein, SMA (superior mesenteric artery) and SMV (superior mesenteric vein). Furthermore, a 12 mm trocar for the assistant surgeon is placed in a periumbilical area (on the left side) [1]. The R1 (first-robotic-arm) is positioned on the left side, from 7 to 10 cm in a lateral position to the assistant surgeon trocar [1]. The R2 (second-robotic-arm) is likewise positioned in the contralateral (right) side. An additional 5 mm assistant surgeon trocar is placed in a location between R2 and the scope port [1]. The R3 (third-robotic-arm) is placed according to the patient body shape (to avert robotic arms collisions). This can be placed far lateral on the left or right side. We do usually prefer to place R3 on the right side, as it helps retracting the pancreatic head laterally during the critical step of uncinate process dissection [1]. When the Xi system is used the 4 robotic ports (8 mm) are positioned in a similar way to the Si-system, but following a straight-line as opposed to a concave line towards the target. Also, the robotic cart placement is more flexible and can be docked in a variable position to meet the OR special requirements [1].

Robotic deployed intra-operative US has definitely a role in the assessment of the lesions, relationship of mesenteric vessels, bile duct anatomy.

#### Step 1: opening of the gastro-colic ligament

The stomach is lifted cranially with the robotic third arm. The lesser sac is entered with hook. The window on the lesser sac is extended with the vessel sealer [1].

#### Step 2: right-colonic-flexure takedown

The hepatic colonic flexure is mobilized with the hook cautery. The mobilization is carried out until full visualization of the duodenum and pancreatic head is achieved [1]. Adequate mobilization also helps in the preliminary identification of the relationship of the middle colic and gastroeiploic vein with the SMV (superior mesenteric vein) [1].

#### Step 3: Kocher maneuver

The third arm retracts the duodeno/pancreatic block laterally and cranially. The Kocher maneuver is carried out in a multistep fashion. Once completed, kocherization should allow visualization of the aorta (left side), the left-renal-vein, and the origin of the SMA (superior mesenteric artery) [1].

#### Step 4: exploration of the hepatic hilum

The liver hilum is dissected to identify the bile duct, the hepatic and gastroduodenal artery and to carry out hilar lymphadenectomy including retroportal lymph nodes. The use of near-infrared indocyanine-green (ICG) fluorescence may be advantageous to confirm the biliary-tree anatomy.[1]. Patients are administered 2.5 mg of ICG 45 min before the beginning of the surgical procedure.

Not all platform have the ICG technology: e.g. should the da Vinci Si model be used, the ICG technology (firefly) must be purchased as an upgrade.

#### Step 5: division of the right-gastric-artery

The right-gastric artery is idenfied, skeletonized, ligated and divided at its origin [1].

#### Step 6: division of the right-gastroepiploic artery

In order to get a proper vascular anatomy visualization and to achieve enough tension, a vertical-cranial-lifting of the antrum of the stomach by R3 (Robotic third arm) is performed. The transection of the gastro-epiploic artery is carried out 1 cm from its origin [1].

#### Step 7: division of the duodenum

When the pylorus preserving technique is chosen, the duodenum is divided 1 cm distally to the pylorus with an endo-stapler [1].

#### Step 8: takedown of the gallbladder

The gallbladder is taken down up to the cystic duct which is not divided in order to perform an en bloc resection [1].

#### Step 9: transection of the common-bile-duct

The CBD (Common Bile Duct) is divided [1].

#### Step 10: transection of the gastro–duodenal-artery (GDA)

The gastroduoduoenal aretry is dissected and stapled when possible. Alternatively, ligation/transfixion with non-absorbable sutures is carried out [1].

#### Step 11: transection of the first-jejunal-loop

The division of the duodeno-jejunal flexure and the rightwards derotatory maneuvers on the duodenum have to be performed before transecting the pancreatic neck. Using R 3, the mesocolon is retracted upwards in order to achieve a better exposure of the Treitz ligament. Harmonic shears (da Vinci Harmonic ACE™ Curved Shears, Intuitive Surgical, Inc) or monopolar hook can be utilized for this step. The main pupose of this maneuver is to reach an extensive mobilization of D4 (4th portion of the duodenum). The first loop of the jejunum is divided by stapler.

Following division of the first-jejunal loop, the mesenteric detachemnt from the jejunum is carried out as far proximal as possible to allow the passage of the duodenum to the right of the aorto-mesenteric axis [1].

#### Step 12: transection of the pancreatic neck

A perfect exposure of the inferior/superior edges of the pancreatic neck is paramount in order to safely perform this step. In order to retract/lift the pancreatic parenchyma and control bleeding from the inferior pancreatic artery, two 3-0 polypropylene stay sutures are placed on the inferior edge of the pancreas. The console surgeon performs the pancreatic neck transection, following the plane anterior to the portal vein using da Vinci Harmonic ACE™ Curved Shears ( Intuitive Surgical, Inc) [1]. A progressively increased tension is exerted on both stay sutures in order to achieve proper retraction while the transection proceeds. Notably, the portal vein/pancreas tunnel is rarely created at once. Rather, it is incrementally obtained while transecting the pancreas parenchyma proceeding safely in the peri-adventitial plane. After the transection of the parenchyma, the duct is cut again with cold scissors to achieve sharp edges and cannulated with a stent (secured applying a 5/0 polydioxanone). An intraoperative frozen section of the pancreatic duct resection margin is obtained.

#### Step 13: the uncinate process dissection

The basic dissection is carried out by the Harmonic shears and the application of non-absorbable polypropylene monofilament stiches for bigger caliber vessels. An “hanging manuever”, which is performed by positioning a vessel-loop around the SMV (superior mesenteric vein) and applying gentle retraction laterally, can be done to optimize the exposure and dissection of the uncinate process especially with regards to its attachments/vessel connection to the SMA (superior mesenteric artery), (meso-pancreas). The dissection is done in a caudal-to-caphalad direction [1].

#### Step 14: pancreatico–gastro/jejunostomy

A small stent (umbilical catheter) of size 2.5 French is placed in the pancreatic duct and anchored with 5/0 PDS. Alternatively, a 5 or 8 French feeding tube can be also used. The posterior capsule of the pancreatic stump is anastomosed to the jejunal serosa via polypropylene stitches. A small opening in the jejunal mucosa is performed and a duct-to-mucosa anastomosis is fashioned with interrupted 4/0 PDS stiches. Alternatively, for larger ducts, two semi-continous sutures can be used [1].

#### Step 15: hepatico–jejunostomy (HJ)-biliary reconstruction

The HJ anastomosis is performed in two layers. The posterior layer is fashioned with a continous suture of 4 or 5-0 PDS. The anterior one is performed with interrupted-PDS-sutures which are placed/secured from slippage with a Hem-o-lok. After the application of all sutures has been completed, these are individually ligated [1].

#### Step 16: pylorus/gastro–jejunostomy (Duodeno–jejunal reconstruction)

This anastomosis is carried out by the use of two 3-0 PDS running sutures. Some polypropilene interrupted stitches can be placed to strengthen the corners and the anterior wall. Near infrared indocyanine-green (ICG) enhanced fluorescence test can be used to evaluate the duodenal stump perfusion [1]. If so, an additional ICG injection is re-administered to the patient (same dose previously specified) to evaluate the arterial perfusion of the anastomotic stumps.

#### Step 17: specimen-extraction and closure

The specimen is positioned in an endobag and extracted by a Pfannenstiel incision [1].

## Discussion

RPD has been increasingly adopted worldwide and in few centers is the preferred approach by certain surgeons. However, the complexity and lack of technique standardization has slowed down its widespread utilization. Some large retrospective series are available and data seem to indicate that RPD is safe/feasible, and a valid alternative to the classic open Whipple [1].

We have previously described a detailed step-by-step RPD guide of our surgical technique [1]. The aim of this complementary project is to facilitate the understanding of our technique with the aid of an educational video. A step-by-step educational approach, complemented with videos, can favor the standardization of the learning process. Our hope is that by standardizing the technique, a faster learning curve can safely be achieved [1, 2] while acquiring and then mastering incrementally more difficult operative maneuvers structurally organized into different standardized surgical steps. The video guidance is a very useful tool in this process.

The Whipple procedure is inherently technically challenging (also in the most “simple” cases), and if it is not performed safely, major intraoperative complication may arise. We have divided our technique in steps to give the possibility to surgeons at the beginning of the learning curve to incrementally/safely acquire (under supervision of a more expert surgeon), all the skills necessary to safely perform the operation. Every step has a different degree of technical complexity, and presents its own technical challenges.

Open and laparoscopic experience does not always directly translate into robotic surgical skills. The robotic learning curve should start with more simple procedures first. The degree of complexity should be progressively increased, and finally (when a reasonable degree of robotic proficiency is achieved) the robotic training on the Whipple procedure should be started. Moreover, at the beginning of the RPD learning curve, less challenging cases should be selected (e.g. small tumors) and every operation should be performed under close supervision of an expert surgeon. Finally, after achieving a safe degree of proficiency, the first cases without supervision should be performed incrementally.

RPD has not yet been accepted as the gold standard for pancreatic head surgery. This is due to the fact that it is a technically demanding surgery and well-powered randomized controlled trials are complex to organize. The literature overall appears to have significant heterogeneity. The validation process is currently mainly bound to be based on retrospective studies. However, large retrospective series are available [3]. At this point, data seem to indicate that RPD is a valid alternative to the classic open Whipple [1, 3–6] and the procedure is safe/feasible in experienced hands [3, 6]. The development of more advanced robotic platforms, alongside the adoption of a standardized technique among different institutions, will conceivably continue to provide additional evidence in this regards. At this stage, in order to further better define the role of RPD, more randomized controlled trial are required [1, 3, 6]. If a new approach is introduced in the clinical setting, a rigorous scientific substantiation, via high level of evidence studies (e.g. RCT and metanalyses), is mandatory [1, 7]. This validation process is a difficult endeavor, the level of technical/operative sophistication is high and the sample sizes required to organize well-powered RCTs are of a considerable order of magnitude. Hence, a multicenter national and international cooperative networks (ideally following a standardized homogenous technique) should be pursued.

## Electronic supplementary material

Below is the link to the electronic supplementary material.

Supplementary file1 (MP4 209351 kb)

## References

[1] Giulianotti PC, Mangano A, Bustos RE (2018). Operative technique in robotic pancreaticoduodenectomy (RPD) at University of Illinois at Chicago (UIC): 17 steps standardized technique lessons learned since the first worldwide RPD performed in the year 2001. Surg Endosc.

[2] Giulianotti PC, Addeo P, Buchs NC (2011). Robotic extended pancreatectomy with vascular resection for locally advanced pancreatic tumors. Pancreas.

[3] Peng L, Lin S, Li Y, Xiao W (2017). Systematic review and meta-analysis of robotic versus open pancreaticoduodenectomy. Surg Endosc.

[4] Ricci C, Casadei R, Taffurelli G, Pacilio CA, Ricciardiello M, Minni F (2017). Minimally invasive pancreaticoduodenectomy: what is the best “choice”? A systematic review and network meta-analysis of non-randomized comparative studies. World J Surg.

[5] Pędziwiatr M, Małczak P, Pisarska M, Major P, Wysocki M, Stefura T, Budzyński A (2017). Minimally invasive versus open pancreatoduodenectomy-systematic review and meta-analysis. Langenbecks Arch Surg.

[6] Kornaropoulos M, Moris D, Beal EW, Makris MC, Mitrousias A, Petrou A, Felekouras E, Michalinos A, Vailas M, Schizas D, Papalampros A (2017). Total robotic pancreaticoduodenectomy: a systematic review of the literature. Surg Endosc.

[7] Mangano A, Rausei S, Lianos GD, Dionigi G (2015). Quality of life after gastrectomy for adenocarcinoma: a prospective cohort study. Ann Surg.

